# Incidents of Animal Life

**Published:** 1887-11-05

**Authors:** F. O. Morris

**Affiliations:** Author of "A History of British Birds," "The Gamekeepers' Museum," and the Humanity Series of School Books


### INCIDENTS OF ANIMAL LIFE.

Collated by the Rev. F. O. Morris, B.A., Author of "A History of British Birds," "The Gamekeepers' Museum," and the Humanity Series of School Books.

"He prayeth best, who loveth best
All things both great and small;
For the dear God who loveth us,
He made and loveth all."—Coleridge.

### A Rival to "Travelling Jack."

"There is (or was till lately) in South Africa a rival to the well-known 'Travelling Jack,' of Brighton line fame, after whom, indeed, he had been nicknamed by his acquaintance. I was introduced to him eighteen months ago on board the *Norham Castle*, on a voyage from Cape Town to England —a voyage which this distinguished Colonial traveller was making much against his will. He was a black-and-tan terrier with a white chest, whose intellect had therefore probably been improved by a dash of mongrelism, and I was told that he belonged to a gentleman connected with the railway department living at Port Elizabeth. It appears that it was 'Mr. Jack's' habit frequently to embark all by himself on board the mail steamer leaving that place on Saturday afternoon, and make the trip round the coast to Cape Town, arriving there on Monday morning. Where he 'put up' I do not know, but he used to stay there until Wednesday evening, when he would calmly walk into the station, take his place in the train, and return to Port Elizabeth in that way, thus completing his 'circular tour' by a railway journey of about eight hundred miles. He was well known by all the officers and sailors of the *Norham*, and her commander, Captain Alexander Winchester (who can vouch for these facts), told me that, as the dog seemed fond of the sea, he had determined to give him a long voyage for a change, and had kept him shut up on board during the ship's stay at Cape Town. 'Jack' was evidently very uneasy at being taken on beyond his usual port, and he was on the point of slipping into a boat for the shore at Madeira, probably with a view of returning to the Cape by the next steamer, when I called the captain's attention to him, and he was promptly shut up again. I said good bye to him at Plymouth, and hope he found his way home safely on the return voyage."

